# P-972. Getting Real *Clean*: Implementation of Virtual Reality Training for Cleaning and Low-Level Disinfection of Portable Medical Equipment

**DOI:** 10.1093/ofid/ofae631.1162

**Published:** 2025-01-29

**Authors:** Michelle S Jerry, Esteban A Barreto, Eileen F Searle, Chloe V Green, Erica S Shenoy

**Affiliations:** Massachusetts General Hospital, Boston, Massachusetts; Massachusetts General Hospital, Boston, Massachusetts; Massachusetts General Hospital, Boston, Massachusetts; Massachusetts General Hospital, Boston, Massachusetts; Mass General Brigham, Boston, MA

## Abstract

**Background:**

Failure to clean and disinfect portable medical equipment (PME) is common, with reported contamination rates between 25% and 100% across a wide range of PME. Virtual reality (VR) is a promising tool for training healthcare personnel (HCP). Despite emerging evidence of its effectiveness as a pedagogical tool, implementation of VR training for infection prevention and control (IPC) and specifically cleaning and low-level disinfection (LLD) of non-critical PME is scarce.

**Table 1:**
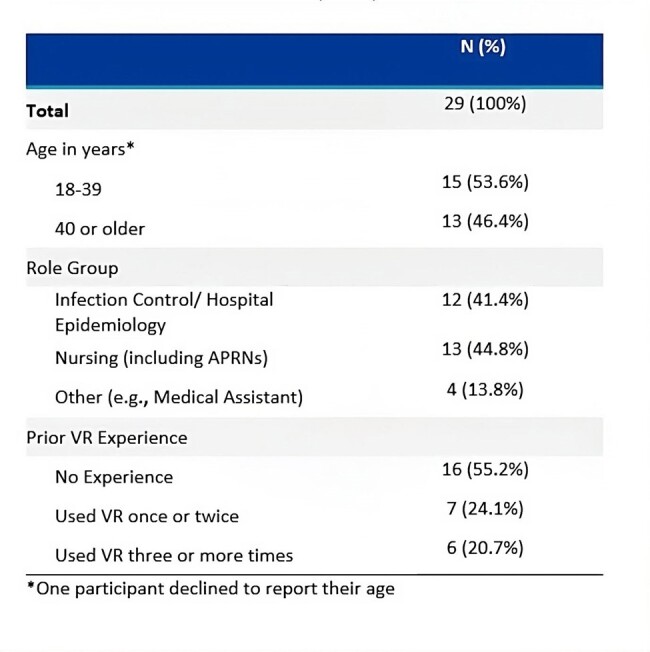
End User Characteristics (N=29).

**Methods:**

A VR training module was developed by subject matter experts in IPC and competency-based education to provide instruction on cleaning and LLD of PME. Convenience sampling design was used to recruit HCP end users. A semi-structured interview guide was developed by qualitative assessment experts to evaluate end user experience. Double coding of verbatim transcripts was independently performed by project staff, with a senior analyst reviewing the analysis.

**Figure 1:**
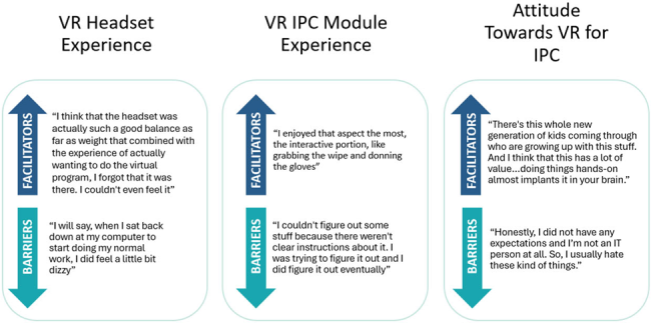
Barriers and Facilitators to Using VR for IPC Training Organized by Theme.

**Results:**

Between January 2024-April 2024, 29 end users from 3 healthcare facilities completed the VR training and semi-structured interviews. More than half (n=16, 55%) did not have prior VR experience (Table 1). Three main themes emerged, including both barriers and facilitators to adoption (Figure 1). End users reported several barriers to their VR Headset Experience, including blurry vision (n=16, 55%); negative physical sensations (n=9, 31%); and difficulty navigating the controllers (n=12, 41%) (Table 2). Despite noted barriers facilitators were identified: end users described the VR IPC Module Experience as “immersive” (n=12, 41%), “fun” (n=8, 28%), and “helpful” (n = 18, 62%). End users' Attitude Towards VR for IPC was overall positive, while noting barriers regarding implementation. End users also identified specific high-value teaching points (Figure 2).

**Table 2:**
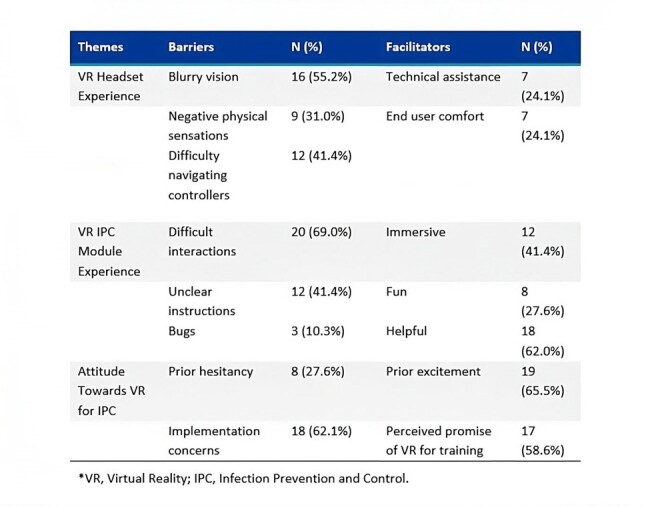
Barriers and Facilitators by Frequency Reported.

**Conclusion:**

While VR training is increasingly used in medical education, efforts to assess its implementation for training in IPC are lacking. This study identified the perceived value of VR training, while highlighting technical and implementation concerns. Future efforts should focus on strategies to mitigate technical and physical sensation concerns, and the evaluation of VR as a teaching modality.

**Figure 2:**
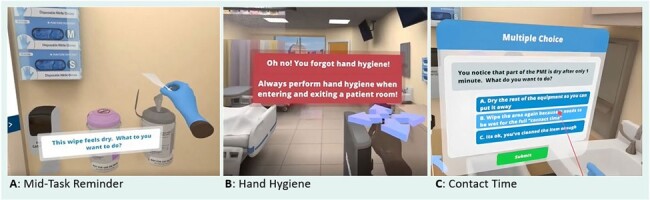
VR Scenarios and Teaching Points End Users Reported as Helpful.

In frame 2A, user draws a disinfectant wipe from the canister and is prompted that the wipe is not wet and asked what they want to do. User then must discard the wipe and get a fresh, wet one, to advance. Teaching point: Wet time/Contact time requires that the product be wet when applied. In frame 2B, if user tries to enter or exit the patient room without performing hand hygiene (either soap and water wash or alcohol-based hand rub), they will be alerted of the missed step. Teaching point: when to perform hand hygiene in the context of the course. In frame 2C, user is alerted that the equipment did not remain wet for full wet time/contact time and is prompted to take action. Teaching point: importance of equipment remaining wet for the defined contact time to achieve disinfection.

**Disclosures:**

**All Authors**: No reported disclosures

